# Geometric morphometric footprint analysis of young women

**DOI:** 10.1186/1757-1146-6-27

**Published:** 2013-07-25

**Authors:** Jacqueline Domjanic, Martin Fieder, Horst Seidler, Philipp Mitteroecker

**Affiliations:** 1 Department of Clothing Technology, University of Zagreb, Zagreb, Croatia; 2 Department of Anthropology, University of Vienna, Vienna, Austria; 3 Department of Theoretical Biology, University of Vienna, Althanstrasse 14, Vienna, A-1090, Austria

**Keywords:** Foot asymmetry, Body mass index, Footprint shape, High heels, Semilandmarks, Shoe design

## Abstract

**Background:**

Most published attempts to quantify footprint shape are based on a small number of measurements. We applied geometric morphometric methods to study shape variation of the complete footprint outline in a sample of 83 adult women.

**Methods:**

The outline of the footprint, including the toes, was represented by a comprehensive set of 85 landmarks and semilandmarks. Shape coordinates were computed by Generalized Procrustes Analysis.

**Results:**

The first four principal components represented the major axes of variation in foot morphology: low-arched versus high-arched feet, long and narrow versus short and wide feet, the relative length of the hallux, and the relative length of the forefoot. These shape features varied across the measured individuals without any distinct clusters or discrete types of footprint shape. A high body mass index (BMI) was associated with wide and flat feet, and a high frequency of wearing high-heeled shoes was associated with a larger forefoot area of the footprint and a relatively long hallux. Larger feet had an increased length-to-width ratio of the footprint, a lower-arched foot, and longer toes relative to the remaining foot. Footprint shape differed on average between left and right feet, and the variability of footprint asymmetry increased with BMI.

**Conclusions:**

Foot shape is affected by lifestyle factors even in a sample of young women (median age 23 years). Geometric morphometrics proved to be a powerful tool for the detailed analysis of footprint shape that is applicable in various scientific disciplines, including forensics, orthopedics, and footwear design.

## Background

The analysis of normal and pathological variation in human foot morphology is central to several biomedical disciplines, including orthopedics, orthotic design, sports sciences, and physical anthropology, and it is also important for efficient footwear design. Genetic factors (including gender) as well as environmental and lifestyle factors (e.g., body weight, shoe wearing habits) have been shown to influence adult foot morphology [1-7]. Human foot shape changes in the course of postnatal development [8] and differs among certain ethnic groups [1,9].

A classic and frequently used approach to study foot morphology is the analysis of the two-dimensional footprint, despite the apparent loss of information along the vertical dimension. Footprints are relatively easy to produce and to measure, and they can be preserved naturally in different soils. In a forensic context, footprint shape can be used in the identification process [2]. Foot print shape is frequently classified into discrete types such as pes planus (flat foot) and pes cavus (high-arched foot) by visual inspection. There have also been proposed a wide range of different quantitative measures and indices of footprint shape, mainly based on the geometry of the medial longitudinal arch. These parameters have been used to create various foot typologies [8,10,11]. Most of these quantifications are based on a small number of characteristics of footprint shape, such as the areas of different parts of the footprint, the curvature of the medial longitudinal arch, or the orientation of the forefoot relative to the rearfoot. However, these measures are insufficient to describe the entire footprint shape and require an a priori selection of the shape features of interest (see [12-16] for more comprehensive approaches).

In the present paper we apply geometric morphometric methods to study the shape of the entire footprint outline in a sample of adult women. Geometric morphometrics (GM) is based on the Cartesian coordinates of landmarks (measurement points) that are homologous across all measured individuals [17-19]. In contrast to a small number of indices, the set of all landmark coordinates preserves the geometry of the measured landmark configurations, and statistical results, such as group means, regressions, or principal components, can thus be represented as actual shapes or shape deformations. Geometric morphometrics is of superior statistical power than most traditional morphometric approaches and is particularly effective for exploratory studies [17-21].

Landmark configurations need to be registered (superimposed) prior to any statistical analysis because the coordinates not only contain information on the shape of the measured objects, but also on their position, scale, and orientation. The most common superimposition technique in geometric morphometrics is Generalized Procrustes Analysis (GPA) [22,23], consisting of three steps. All landmark configurations are (i) translated to have the same centroid (average landmark position), (ii) scaled to have the same size, and (iii) iteratively rotated to minimize the summed squared distances between the landmarks and the corresponding sample average. Overall size is measured as Centroid Size, the square root of the summed squared distances between the landmarks and their centroid [17]. Procrustes registration is based on all landmarks and on their explicit correspondence (homology) across specimens. It does not require the specification of reference points or lines and is more stable than simple principal component alignment (For maximum-likelihood based versions of Procrustes registration see [24,25]). The coordinates of the superimposed landmark configurations are called Procrustes shape coordinates as they contain information about the shape of the landmark configurations only. They are the basis for further statistical analysis. Procrustes distance is a measure of shape difference between two objects and is approximated by the Euclidean distance between the two sets of shape coordinates.

Many biological structures, such as footprint outlines, consist of relatively smooth curves and lack homologous landmark points that can be identified in all individuals. Semilandmarks are points along such smooth outlines that are initially placed at approximately corresponding positions; their exact locations are then estimated statistically in order to create geometrically homologous landmarks that can be used in the subsequent analysis as if they were anatomical landmarks. The most common algorithm for this purpose is the sliding landmark algorithm [26,27], which iteratively slides the semilandmarks along their curves in order to minimize local shape differences (the bending energy of the thin-plate spline interpolation) between each individual and the sample average.

In the present study, we extracted the footprint shape from three-dimensional surface scans of the feet (see the methods section below), but geometric morphometric methods can also be applied to other techniques for capturing footprints, such as ink footprints or pressure platforms.

## Methods

The feet of 83 female individuals, aged between 19 and 36 years (median age 23 years), were scanned with a “Pedus” laser foot scanner (Vitronic and Human Solutions GmbH, Germany), located in the Department of Clothing Technology at the University of Zagreb. A total of four scans were made for each person, two of the left foot and two of the right foot. Additionally, age, body weight, body height, shoe size, sports activities, shoe wearing habits, and handedness were recorded for each person. According to their place of birth, the women were grouped into four geographic categories: the continental (*N*=45), the Adriatic (*N*=20), and the Slavonian region of Croatia (*N*=8), as well as a group of women from other countries (Bosnia and Herzegovina, Kosovo, France, and Austria; *N*=10). Participation in the survey was entirely voluntary and based on written consent. The study was approved by the Ethics Committee of the Faculty of Textile Technology, University of Zagreb, on July 18th, 2012.

**Figure 1:**
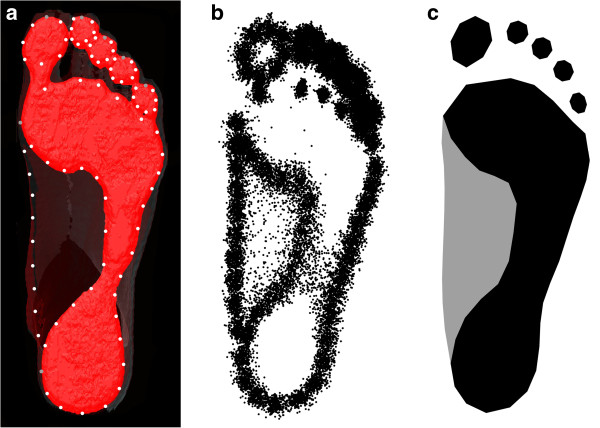
**Landmark scheme for measuring footprint shape. (a)** The footprint of one individual, extracted from a three-dimensional surface scan, together with the landmarks and semilandmarks used for the morphometric analysis shown as gray and white points. **(b)** Landmark configurations of all individuals after Procrustes superimposition. **(c)** Visualization of the average footprint shape (average shape coordinates) in the sample.

Using the software Amira (Imersion Inc.), the scanned surfaces were rendered and the footprints were extracted by cutting off the lowest (plantar-most) 2mm of the foot scan. The opacity of the remaining scan was reduced so that the full outline of the foot was still visible. After exporting a screenshot of the footprint and the foot outline, 85 landmarks and semilandmarks (Figure 1a) were digitized using the software TPSdig 2.0 (James Rohlf). For each toe, the outline of the distal element was digitized by 7 semilandmarks and 1 anatomical landmark at the distal-most tip. The outline of the remaining footprint, including the footpad, the midfoot, and the heel, was digitized by 34 semilandmarks and two anatomical landmarks at the two medial-most positions. The medial outline of the foot (starting and ending at the medial-most positions of the footprint outline) was digitized by 9 semilandmarks.

We used the sliding landmark algorithm [26] to estimate the position of the semilandmarks in all individuals, enabling the joint analysis of anatomical landmarks and curves (represented by semilandmarks). All landmark configurations were superimposed by a Generalized Procrustes Analysis [22], standardizing for position, size, and orientation of the configurations. The resulting Procrustes shape coordinates (Figure 1b) were used for further statistical analysis.

We performed a principal component analysis (PCA; also referred to as relative warp analysis by [17]) of these shape coordinates to investigate the major components of variation in footprint shape. The first principal component (PC) is the shape pattern (linear combination of shape coordinates) with maximum variance in the sample. It can be visualized as a shape deformation or a series of shapes, and a score along the PC can be computed for each individual. The second PC is geometrically orthogonal (perpendicular) to the first one and accounts for the second most variance, and similarly for all subsequent components.

We further assessed the influence of body mass index (body weight divided by squared body height), shoe size, frequency of wearing high heels, age, and sports activities on footprint shape by multivariate regressions of the shape coordinates on the respective variable. For these analyses we averaged the two right footprints of each person with the two mirrored left footprints so that every person was represented by a single symmetric footprint shape. Footprint asymmetry, i.e., the shape differences between left and right footprints, were studied by comparing the right footprints with the reflected left footprints [17-20,28,29]. Levels of statistical significance were computed by permutation tests, using 5000 random permutations. Permutation tests do not require normally distributed variables and can be applied to multivariate datasets that are not of full rank, such as Procrustes shape coordinates [30].

## Results

**Figure 2:**
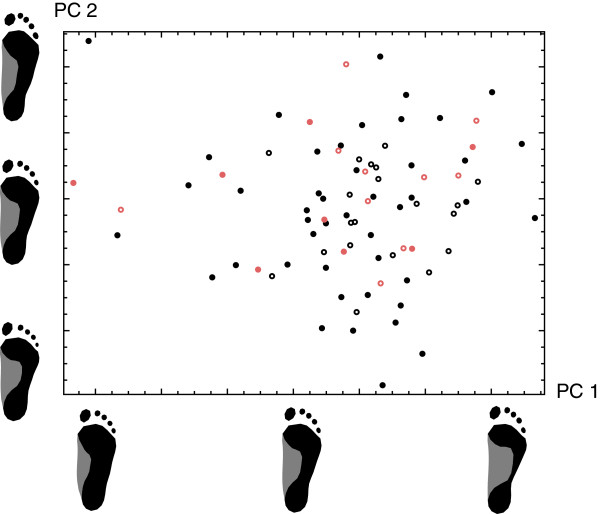
**Scatterplot of the first two principal components of footprint shape.** The first principal component (visualized by the footprint shapes along the PC 1 axis) is a contrast between flatfeet (low PC 1 sores) and high-arched feet (high PC 1 scores), whereas PC 2 (visualized by the footprint shapes along the PC 2 axis) represents the differences between short and wide feet with short toes (low PC 2 scores) versus long and narrow feet with long toes (high PC 2 scores). The symbol type reflects the geographical origin of the individuals: the continental (filled black circles), the Adriatic (open black circles), and the Slavonian region of Croatia (filled red circles), as well as other countries (open red circles).

**Figure 3:**
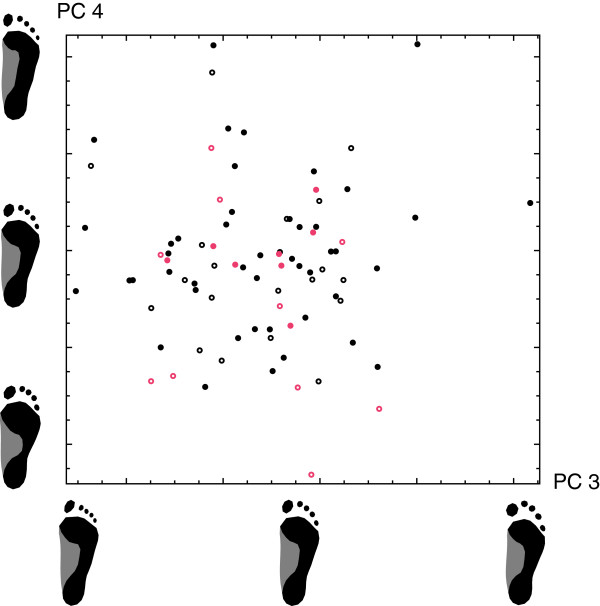
**Scatterplot of principal components 3 and 4 of footprint shape.** The principal components are visualized by footprint shapes along the two corresponding axes. Individuals with a low score along PC 3 have a long and narrow hallux relative to the other toes (“Egyptian foot”), whereas individuals with a high score have a long second toe relative to the hallux (“Greek foot”). PC 4 mainly reflects the relative length of the forefoot. The symbol type corresponds to the geographical origin of the individuals.

The first four principal components (PCs) accounted for 60.3% of total shape variation and are visualized in Figures 2 and 3. PC 1 was a contrast between flatfeet (pes planus; low PC 1 sores) and high-arched feet (pes cavus; high PC 1 scores), whereas PC 2 represented the differences between short and wide feet with short toes (low PC 2 scores) versus long and narrow feet with long toes (high PC 2 scores). PC 3 reflected variation in the length and the shape of the toes. Individuals with a low score along PC 3 had a long and narrow hallux relative to the other toes (sometimes referred to as “Egyptian foot”), whereas for individuals with high scores the second toe was longer than the hallux (“Greek foot”). PC 4 mainly reflected the relative length of the forefoot.

Every symbol in the PCA plots represents the footprint shape of one person. The four different geographic groups (reflected by the symbol type) had a similar distribution along the PCs. Furthermore, pairwise permutation tests did not reveal any significant differences in mean shape between the geographic groups (*p*>0.16 for all tests).

**Figure 4:**
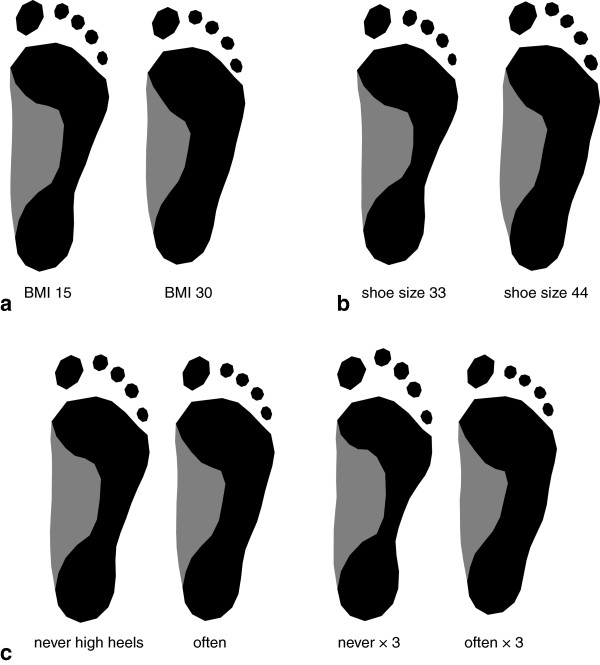
**Visualization of the effects of body mass index (BMI), shoe size, and the frequency of wearing high heels on footprint shape, estimated via linear regressions of shape on the corresponding factor.** The displayed footprints are extrapolations of the actually occurring range of variability in order to effectively visualize the patterns of shape difference. **(a)** Expected footprint shapes for BMI 15 and BMI 30. **(b)** Expected footprint shapes for shoe size 33 and shoe size 44. **(c)** Average footprint shape for women never wearing high heels and for women often wearing high heels, together with footprint shapes derived from a threefold extrapolation of these effects.

We investigated the influence of different factors on footprint shape by regressing the shape coordinates on the respective variables (Figure 4). Body mass index (BMI) had a significant effect on footprint shape (*p*<0.001) and explained 2.8% of total shape variation. A low BMI was associated with a more arched foot and a high BMI with a flatter foot. BMI was further associated with the relative width (length-to-width-ratio) of the foot. Shoe size (measuring foot length) was significantly related to footprint shape (*p*<0.001) and accounted for 2.4% of shape variation. Feet with a larger shoe size tended to have an increased length relative to the width, a lower-arched foot shape, and longer toes relative to the remaining foot. The frequency of wearing high heels significantly explained 1.8% of total shape variation (*p*=0.003). People often wearing high heels tended to have a relatively longer forefoot and a more anterior positioned hallux relative to the other toes. We further analyzed the effect of age and sports activities on footprint shape but found no significant relationships (*p*>0.26).

**Figure 5:**
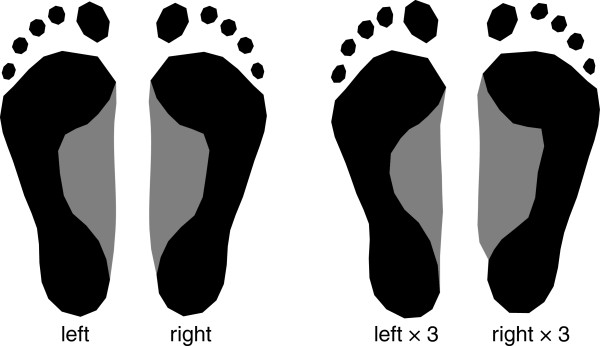
**Average left and average right footprint shape, together with footprint shapes derived from a threefold extrapolation of these differences.**

For all the above analyses, left and right footprints were averaged for each individual, but they can also be analyzed separately in order to investigate shape asymmetry. Figure 5 shows the average left footprint and the average right footprint (differing significantly at *p*<0.001), along with extrapolations of the shape differences between them. Right footprints on average had a slightly increased width relative to the length, and the border between the forefoot and the midfoot was more angulated in right feet than in left ones. We also analyzed footprint shape asymmetry separately for left-handed and for right-handed persons but found no significant difference.

In addition to the average *pattern* of asymmetry, we further quantified the *amount* of asymmetry for every individual as the Procrustes distance between the left and the right foot. While the average amount of asymmetry was not significantly related to any of the factors, the variance of asymmetry was significantly lower for women with BMI<21 as compared to women with BMI>21 (*p*=0.006).

For every person two scans of the left foot and two scans of the right foot were made and digitized with landmarks, so we could also compute the repeatability of the shape variables. The intraclass correlation coefficients (ICC) for the first four principal components were 0.95, 0.85, 0.85, and 0.88, respectively. Note that these ICC coefficients reflect the repeatability of the actual footprint (the way how people stand and how the foot deforms under pressure), of the 3D surface scan and the virtual extraction of the footprint, and of the landmark measurements.

## Discussion

We applied geometric morphometric methods to study variation of footprint shape in a sample of young adult women. The outline of the footprint, including the toes, was represented by a comprehensive set of landmarks and semilandmarks, allowing for a detailed morphological analysis without any prior selection of shape features. The first four principal components of footprint shape – the major axes of variation – represented crucial aspects of foot morphology: low-arched versus high-arched feet, long and narrow versus short and wide feet, the relative length of the hallux, and the relative length of the forefoot. These shape features varied independently across the measured individuals without any distinct clusters or discrete types of footprint shape. The distinction between different foot types, which is very common in the literature [10], hence remains partly arbitrary: the definition of foot types cannot be based entirely on biological variation, but must be designed for specific purposes, such as shoe production or clinical treatment. Different typological systems based on different criteria are unlikely to match.

We investigated the influence of several lifestyle factors on footprint shape. A high BMI was associated with wide and flat feet, which was also found by other researchers. For example, Ashizawa *et al*. [1] and Mauch *et al*. [6] reported an increase of relative foot width with body weight.

A high frequency of wearing high-heeled shoes was associated with a larger forefoot area of the footprint and a hallux exceeding the other toes in length. Heel elevation leads to increased pressure and shear stress on the forefoot, particularly on the medial forefoot [31,32], and several studies reported that older women who frequently wore high heels had an increased prevalence of hallux valgus [33,34]. Since our sample consists of young women (median age 23 years), it is particularly surprising that we found a significant association between foot shape and shoe wearing habits already in this age range.

Right feet on average were slightly wider than left feet and the outline of the medial longitudinal arch was more angulated in right feet. Using elliptic Fourier analysis Sforza *et al*. [13] reported a similar average shape difference between left and right footprints. We also found that the sample variance of the amount of asymmetry increases with BMI. A higher body weight might more easily transform asymmetries of gait and behavior into morphological asymmetries.

Larger feet (measured by shoe size) tend to have an increased overall length *relative* to the width (length-to-width ratio), a lower-arched foot, and longer toes relative to the remaining foot. Such an association of overall size and shape is referred to as allometry [17,23]. This has profound consequences for shoe design: shoes differing in size should also differ in shape, and shoes should not be perfectly symmetric but should reflect the asymmetries of the foot. We did not find differences in average foot shape between the geographic regions covered by our sample.

By applying geometric morphometrics to a comprehensive set of landmarks and semilandmarks along the footprint outline, we were able to assess variation in footprint shape at a very fine spatial scale. We could confirm well-known patterns of shape variation, such as variation in the curvature of the medial longitudinal arch or in the size and orientation of the forefoot relative to the rearfoot, without specifying these patterns prior to the analysis by selecting corresponding measurements. We were thus also able to discover novel patterns, e.g., details in the medial longitudinal arch shape and in the relative size and shape of the toes. The convenient statistical properties of geometric morphometrics together with the effective visualization resulting from the large number of landmarks allow for very powerful exploratory studies in various scientific disciplines, including orthopedics, forensics, and footwear production. The manual measurement protocol used in the current study might be too time-consuming for daily clinical routine, but it is a powerful tool for scientific research and for the generation and evaluation of simple indices of footprint shape. Geometric morphometrics of footprint shape might be used complementary to plantar pressure analysis [35], which typically aims at standardizing for shape variation instead of analysing it.

## Conclusion

We identified the major patterns of variation in footprint shape and estimated the effects of BMI, foot size, shoe wearing habits, and asymmetry on foot morphology. Geometric morphometrics proved to be a powerful tool for assessing the shape of the complete footprint outline. It should be the method of choice for scientific research and for the evaluation of simple indices of footprint shape.

## Competing interests

The authors declare that they have no competing interests.

## Authors’ contributions

JD carried out the surface scans, extracted the footprints from the surface scans, digitized the landmarks, and participated in the data analysis and the drafting of the manuscript. MF participated in the data collection and the design of the study. HS participated in the design and coordination of the study and helped to draft the manuscript. PM analyzed the data, wrote the manuscript, and contributed to the design of the study. All authors read and approved the final manuscript.
